# Isolation of Platelet-Derived Exosomes from Human Platelet-Rich Plasma: Biochemical and Morphological Characterization

**DOI:** 10.3390/ijms23052861

**Published:** 2022-03-05

**Authors:** Miquel Saumell-Esnaola, Diego Delgado, Gontzal García del Caño, Maider Beitia, Joan Sallés, Imanol González-Burguera, Pello Sánchez, Maider López de Jesús, Sergio Barrondo, Mikel Sánchez

**Affiliations:** 1 Department of Pharmacology, Faculty of Pharmacy, University of the Basque Country UPV/EHU, 01006 Vitoria-Gasteiz, Spain; miquel.saumell@ehu.eus (M.S.-E.); maider.lopez@ehu.eus (M.L.d.J.); sergio.barrondo@ehu.eus (S.B.); 2 Bioaraba, Neurofarmacología Celular y Molecular, 01008 Vitoria-Gasteiz, Spain; gontzal.garcia@ehu.eus (G.G.d.C.); imanol.gonzalezb@ehu.eus (I.G.-B.); 3 Advanced Biological Therapy Unit, Hospital Vithas Vitoria, 01008 Vitoria-Gasteiz, Spain; diego.delgado@ehu.es (D.D.); maider.beitia@ucatrauma.com (M.B.); pello.sanchez@ucatrauma.com (P.S.); 4 Department of Neurosciences, Faculty of Pharmacy, University of the Basque Country UPV/EHU, 01006 Vitoria-Gasteiz, Spain; 5 Centro de Investigación Biomédica en Red de Salud Mental (CIBERSAM), 28029 Madrid, Spain; 6 Arthroscopic Surgery Unit, Hospital Vithas Vitoria, 01008 Vitoria-Gasteiz, Spain

**Keywords:** platelet-rich plasma, human platelets, platelet-derived-exosomes, exosome markers, cytokines, growth factors

## Abstract

Platelet-Rich Plasma (PRP) is enriched in molecular messengers with restorative effects on altered tissue environments. Upon activation, platelets release a plethora of growth factors and cytokines, either in free form or encapsulated in exosomes, which have been proven to promote tissue repair and regeneration. Translational research on the potential of exosomes as a safe nanosystem for therapeutic cargo delivery requires standardizing exosome isolation methods along with their molecular and morphological characterization. With this aim, we isolated and characterized the exosomes released by human PRP platelets. Western blot analysis revealed that CaCl_2_-activated platelets (PLT-Exos-Ca^2+^) released more exosomes than non-activated ones (PLT-Exos). Moreover, PLT-Exos-Ca^2+^ exhibited a molecular signature that meets the most up-to-date biochemical criteria for platelet-derived exosomes and possessed morphological features typical of exosomes as assessed by transmission electron microscopy. Array analysis of 105 analytes including growth factors and cytokines showed that PLT-Exos-Ca^2+^ exhibited lower levels of most analytes compared to PLT-Exos, but relatively higher levels of those consistently validated as components of the protein cargo of platelet exosomes. In summary, the present study provides new insights into the molecular composition of human platelet-derived exosomes and validates a method for isolating highly pure platelet exosomes as a basis for future preclinical studies in regenerative medicine and drug delivery.

## 1. Introduction

For more than a decade, intra-articular delivery of autologous Platelet-Rich Plasma (PRP) has emerged as a safe and promising biotechnological alternative for the treatment of different pathologies [1]. PRP is a plasma product from patients’ own blood, which aims to increase the number of platelets and the concentration of molecular mediators that exert therapeutic effects, while eliminating unwanted elements such as red blood cells [2]. Thus, the selective enrichment in growth factors and anti-inflammatory cytokines is considered to be responsible for the effects of PRP in improving clinical conditions in a variety of disease situations [3,4,5,6].

It is well established that exocytosis of granule components from activated platelets is a major source of these molecular mediators [7,8]. In recent years, exosomes isolated directly from PRP (PRP-derived exosomes, PRP-Exos), which are highly enriched in platelet-derived exosomes but also include exosomes from other different cellular sources, or exosomes obtained from washed platelets (platelet-derived exosomes, PLT-Exos) have attracted increasing attention as potential mediators of the effects of PRP and platelet lysates in tissue regeneration [9]. In fact, there is evidence that PLT-Exos are rich in the molecular mediators responsible for the healing effect of PRP [10]. Indeed, human PLT-Exos have been shown to increase cell proliferation and migration of mesenchymal stromal cells (MSCs) from human bone marrow [10], to prevent apoptosis-caused glucocorticoid-associated endoplasmic reticulum stress in a rat model of osteonecrosis [11] and to promote re-epithelization of chronic ulcers in a diabetic rat model [12]. In turn, homologous PRP-Exos have shown to promote proliferation and inhibit apoptosis of rabbit chondrocytes [13] and to promote the formation of vessel-like structures from cultured human umbilical vein endothelial cells while increasing their proliferation and migration rates [14].

Thanks to their unique features, such as high biocompatibility as well as low cytotoxicity, tumorigenicity, and immunogenicity [15,16], PLT-Exos could present advantageous therapeutic properties, including homologous administration in the clinical setting, thus overcoming the restrictive requirement of other biological products. Furthermore, in comparison with other cell sources and fluids used successfully to obtain exosomes [17], platelets allow obtaining higher amounts using minimally invasive procedures. Also noteworthy, PLT-Exos are presumed to cross highly selective biological barriers such as the blood–brain barrier, as has been shown for exosomes from other sources [18], and can be easily administered by a variety of non-invasive routes to increase bioavailability depending on the purpose. For instance, intranasally administered exosomes in animal models have been shown to rapidly reach the brain parenchyma and proven to exhibit biological effects [18,19]. Beyond their direct therapeutic potential, PLT-Exos constitute a promising biotechnological alternative for the delivery of drugs [20] or biomolecules of therapeutic interest. In fact, drugs and biomolecules can be directly loaded into exosomes by means of a variety of strategies [21]. Moreover, phase I clinical trials have been conducted using autologous dendritic cell-derived exosomes loaded with melanoma-associated antigens in metastatic melanoma [22] and advanced non-small cell lung cancer patients [23], and with autologous ascites derived exosomes loaded with granulocyte–macrophage colony-stimulating factor in colorectal cancer patients [24]. All of them ensured feasibility in exosome production and safety in their administration.

Despite the growing body of evidence supporting the potential of exosomes as an attractive and safe biotechnological alternative in the field of advanced PRP-based therapeutics and drug delivery, the standardization of methods for exosome isolation and preparation along with their characterization are unavoidable prerequisites for conducting translational studies. In this sense, the methodological variability in the different steps for the isolation of PLT-Exos can influence the outcome concerning the yield, purity, and specific cargo composition [20]. Indeed, reported methods vary considerably, ranging from the direct purification from activated PRP, under the assumption that most of the exosomes present in PRP are of platelet origin [13,14], to isolating them from lysates of non-activated platelets [10] or from supernatants of activated platelets that have previously been separated from PRP [11,12,25].

With these considerations in mind, the main aim of the present study was to provide updated information on the molecular and morphological characterization of exosomes released from human platelets. To this end, we isolated and characterized by biochemical and morphological criteria highly purified human PLT-Exos released by calcium-activated platelets, which were isolated from PRP obtained from donors and prepared by a method widely proven for its efficacy in vitro [5], in vivo [6], and in clinical studies [26].

## 2. Results

### 2.1. PRP Characterization

The PRP used in this study presents a concentration of platelets 2-fold higher than in blood, with no leukocytes or erythrocytes (Table 1). According to the latest coding system and minimum reporting requirements for PRP studies, the type of PRP was 13-00-11 [27].

### 2.2. Total Protein Content Measurement in Platelets and Platelet-Derived Exosomes under Basal Conditions and Calcium-Stimulation

**Figure 1:**
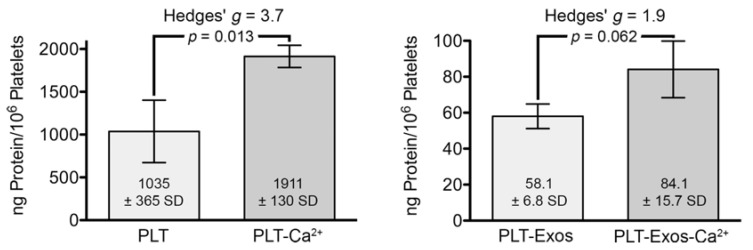
Bar graphs representing the amount of protein, determined by the Bradford method and normalized to 10^6^ platelets, in samples of platelets under basal (PLT) and calcium-stimulated (PLT-Ca^2+^) conditions and in samples of platelet-derived exosomes isolated from platelets at baseline (PLT-Exos) and after stimulation with calcium (PLT-Exos-Ca^2+^). Values shown correspond to mean values ± standard deviation. *p* values were calculated by Student’s unpaired (one-tail) *t*-test (PLT and PLT-Ca^2+^, *n* = 2; PLT-Exos and PLT-Exos-Ca^2+^, *n* = 3). Effect size was estimated by Hedges’ *g*.

Prior to characterizing the purity of exosome samples using non-exosome and exosome-specific markers, we determined the amount of protein in pelleted platelet samples after incubation for 30 min at 37 °C in PBS in the absence (PLT) or presence (PLT-Ca^2+^) of CaCl_2_ (10 µL CaCl_2_ 10%-*w*/*v*-per mL PRP) and in exosomes isolated from them (PLT-Exos and PLT-Exos-Ca^2+^, respectively). The data, expressed as nanograms of protein per million platelets contained in the initial volume of PRP used to isolate the samples, showed that calcium stimulation led to a significant increase in the amount of platelet protein (*p* = 0.013) and a trend toward an increase in PLT-Exos-Ca^2+^ relative to PLT-Exos, approaching statistical significance (*p* = 0.062) at the 95% confidence interval by Student’s *t*-test (Figure 1). This result, together with the large effect size as reflected by a Hedges’ *g* of 1.9, indicates that calcium prompted exosome release from platelets.

### 2.3. Biochemical Characterization of Human Platelet-Derived Exosomes Obtained under Basal Conditions and Calcium-Stimulation

**Figure 2:**
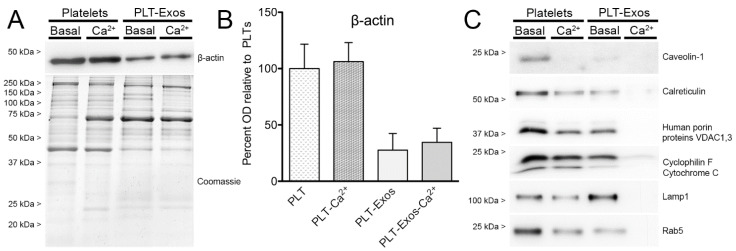
Western blot analysis of non-exosome components in samples of human platelets and platelet-derived exosomes. (**A**) Protein loading controls by immunoblotting using an antibody against the cytoskeletal protein β-actin and by Coomassie Blue-staining of total proteins in platelet (PLT) samples, either non-activated (basal) or calcium-activated (Ca^2+^), and in platelet-derived exosomes (PLT-Exos) isolated from platelets at baseline or after stimulation with calcium. The immunoblot for β-actin corresponds to one of the two independent assays performed on samples from one volunteer. Coomassie blue staining was performed on samples pooled from volunteers 1 and 2. (**B**) Bar graph depicts the mean optical density values of immunoreactive bands for β-actin in the different samples relative to non-activated platelets. PLT, non-activated platelets; PLT-Ca^2+^, calcium-activated platelets; PLT-Exos, exosomes isolated from non-activated platelets; PLT-Exos-Ca^2+^, exosomes isolated from calcium-activated platelets. Data are mean ± SD of two independent experiments performed on samples from volunteers 1 and 2 (*n* = 2). (**C**) Immunoblot against proteins of non-exosome cell components (Table 2, for details) in PLT, PLT-Ca^2+^, PLT-Exos, and PLT-Exos-Ca^2+^ samples pooled from volunteers 1 and 2.

Success in exosome purification from platelets under basal and calcium-stimulation conditions was assessed by Western blot analysis on PLT, PLT-Ca^2+^, PLT-Exos, and PLT-Exos-Ca^2+^ samples. To this end, equal amounts of each of the four samples were resolved side by side in SDS-polyacrylamide gels (SDS-PAGE). As protein loading controls, we used immunoblotting for β-actin and Coomassie blue staining. No differences in β-actin immunoreactivity were observed between PLT versus PLT-Ca^2+^ and PLT-Exos versus PLT-Exos-Ca^2+^ samples, and the intensity of Coomassie staining was similar among the four samples. However, differences in the band patterns of Coomassie-stained proteins could be clearly observed, both between platelet and exosome samples and between the two platelet samples and the two exosome samples compared with each other (Figure 2A,B), indicating that calcium affects the expression of platelet proteins and the release of protein cargo to platelet-derived exosomes.

**Figure 3:**
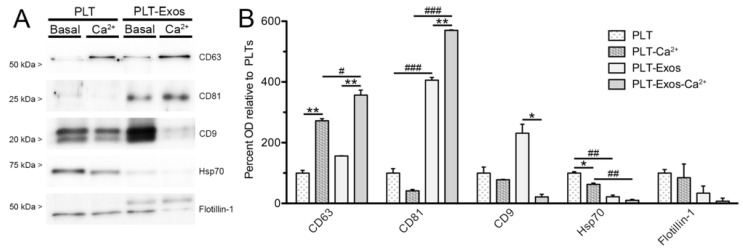
Biochemical analysis of the enrichment of platelet-derived exosome samples in exosome-specific markers. (**A**) Immunoblot against exosome-specific markers (see Table 2 for details) in platelet (PLT) samples, either non-activated (basal) or calcium-activated (Ca^2+^), and in platelet-derived exosomes (PLT-Exos) isolated from platelets at baseline or after stimulation with calcium. The immunoblot corresponds to one of the two independent assays performed on samples from one volunteer. (**B**) Bar graph depicts the mean optical density values of immunoreactive bands for five antigens widely used as exosome markers in the different samples relative to non-activated platelets. PLT, non-activated platelets; PLT-Ca^2+^, calcium-activated platelets; PLT-Exos, exosomes isolated from non-activated platelets; PLT-Exos-Ca^2+^, exosomes isolated from calcium-activated platelets. Data are mean ± SD of two independent experiments performed on samples from volunteers 1 and 2 (*n* = 2). Asterisks (*) and hashes (#) refer to comparisons between basal and calcium-activation conditions for each of the samples (PLT versus PLT-Ca^2+^, PLT-Exos versus PLT-Exos-Ca^2+^) and between exosomes and platelets of the same condition (PLT versus PLT-Exos and PLT-Ca^2+^ versus PLT-Exos-Ca^2+^). * *p* < 0.05, ** *p* < 0.01, # *p* < 0.05, ## *p* < 0.01, ### *p* < 0.005. *p* values were calculated by one-way repeated measures ANOVA with Bonferroni post hoc test.

To analyze the presence of particles of non-exosome origin in PLT-Exos and PLT-Exos-Ca^2+^ samples, we carried out Western blot assays using antibodies against cell surface (caveolin-1), mitochondrial (human porin proteins VDAC 1 and 3, cyclophilin F/cytochrome C), endoplasmic reticulum (calreticulin), lysosomal (lamp1), and early endosome proteins (Rab5). The results revealed that PLT-Exos exhibited some degree of contamination with all the non-exosome cell components, whereas immunoreactivity was undetectable or barely above detection threshold for all the antigens analyzed in PLT-Exos-Ca^2+^, indicating that exosomes isolated from calcium-activated platelets were of high purity. Nevertheless, immunoreactivity for these non-exosome markers was more intense in PLT-Exos than in the PLT samples from which they were isolated (Figure 2C), leaving open the possibility that particles isolated from non-activated platelet supernatant might still be rich in exosomes. Indeed, both PLT-Exos and PLT-Exos-Ca^2+^ exhibited clear immunoreactivity for several markers classically considered as exosome-specific, although with obvious differences between the two samples in signal levels for the different antigens (Figure 3A). Thus, in relation to the platelets used as the source for their isolation, PLT-Exos were enriched for CD81 and CD9 (but not CD63), whereas PLT-Exos-Ca^2+^ were enriched for CD63 and CD81 (but not CD9). As a consequence of these differences, PLT-Exos-Ca^2+^ exhibited significantly higher levels of CD63 and CD81 than PLT-Exos and, conversely, lower levels of CD9. Moreover, in PLT-Exos-Ca^2+^, CD9 levels showed a strong decreasing trend compared to calcium-activated platelets. Unlike what was observed for the tetraspandins CD63, CD81, and CD9, the levels of the chaperone Hsp70 were significantly lower in both exosome samples with respect to their corresponding platelet sources, and a similar trend was observed for the lipid raft marker flotillin-1 (Figure 3B).

### 2.4. Analysis of Cytokines, Chemokines, and Growth Factors in PPP, PRP, PLT-Exos, and PLT-Exos-Ca^2+^

Semiquantitative measurement of 105 analyte levels, including cytokines, chemokines, growth factors, and other soluble proteins, was performed side by side in samples of human Platelet-Poor Plasma (PPP) and CaCl_2_-activated PRP (PRP), and in PLT-Exos or PLT-Exos-Ca^2+^ washed from human PRP.

Comparison between the PPP and PRP samples showed a general trend for analyte levels to increase in PRP relative to PPP. Thus, PRP showed significantly higher levels of 12 analytes with respect to PPP, while only six decreased. Out of the five analytes found to increase more than two-fold in PRP in relation to PPP, 4 corresponded to growth factors (EGF, BDNF, PDGF-AA, and PDGF-AB/BB) and one to the CXCL5 chemokine.

**Figure 4:**
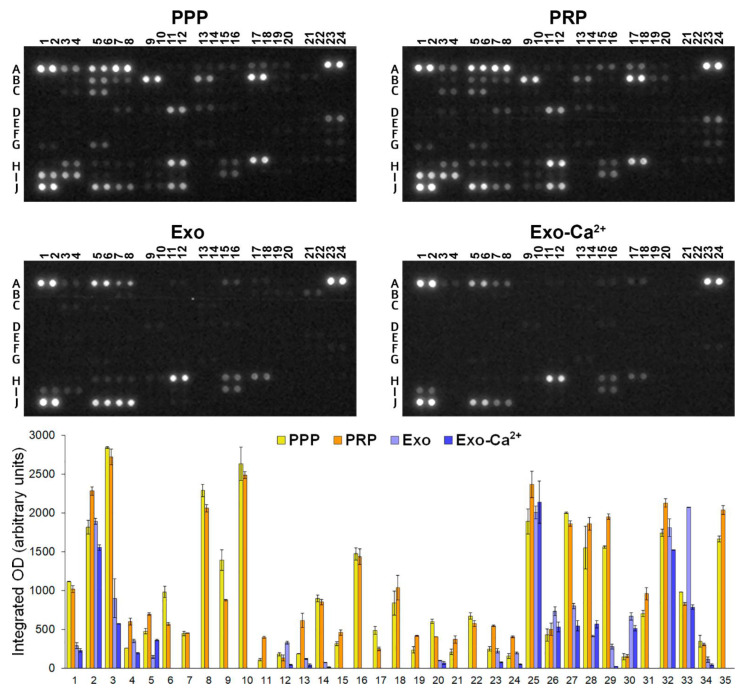
Analysis of cytokines, chemokines, and growth factors in samples of platelet-poor plasma (PPP), platelet lysate from activated platelet-rich plasma (PRP), exosomes isolated from non-activated platelets (Exo), and exosomes isolated from CaCl_2_-activated platelets (Exo-Ca^2+^). The numbers and letters in antibody arrays at the top of the figure depict coordinates corresponding to the different analyte duplicates shown in Table 2. The bar graph at the bottom of the figure shows integrated optical density (OD) values (mean ± SD) measured in duplicate (two spots per analyte). The correspondence between the numbers in abscissa and analytes corresponding to the different coordinates are shown in Table 2.

As a first overview of the data obtained from exosome samples, it was observed that many of the analytes detected in PPP and PRP samples had significantly lower levels or were undetectable in both PLT-Exos and PLT-Exos-Ca^2+^. However, it is important to consider that 140 times more plasma protein (PPP and PRP, 10.5 mg) than exosome protein (PLT-Exos and PLT-Exos-Ca^2+^, 75 μg) was added to the assay. On the other hand, considering the extremely low proportion of protein in purified PLT-Exos and PLT-Exos-Ca^2+^ relative to total PRP protein (0.19 ± 0.05 and 0.26 ± 0.08%, respectively), our data show that platelet-derived exosomes contribute negligibly to the analyte levels detected in the PPP and PRP samples. Indicative of successful exosome separation from PRP, the signal intensities in PLT-Exos and PLT-Exos-Ca^2+^ differed markedly from that observed in PPP and PRP samples from the same volunteers. As the most extreme representative examples of this, several analytes whose signals were among the strongest in PRP were undetectable (C-reactive protein, dipeptidyl peptidase-4 [DPP4], IGFBP-2, IL18-binding protein, Thrombospondin-1, VCAM-1) or showed a very low signal (SHBG) in PLT-Exos and PLT-Exos-Ca^2+^. In contrast, other analytes exhibited nearly equal (Platelet factor 4, CCL5, VDB) and even higher (TFF3, PECAM-1) levels in exosome samples relative to PRP. When the signals produced by exosomes derived from non-activated and calcium-activated platelets were compared with each other, it was evident that the signal from most of the 20 detected analytes was lower in exosomes isolated from activated platelets. Specifically, statistically significant lower levels were found for 10 analytes and a similar not statistically significant trend for another 7, whereas only two molecules showed (Complement C5/C5a) or hovered around (PAI-1, *p* = 0.051) statistically significant higher levels in PLT-Exos-Ca^2+^ than in PLT-Exos (Figure 4, Table 2).

### 2.5. Transmission Electron Microscopy Analysis of Exosomes Isolated from Calcium-Activated Platelets

**Figure 5:**
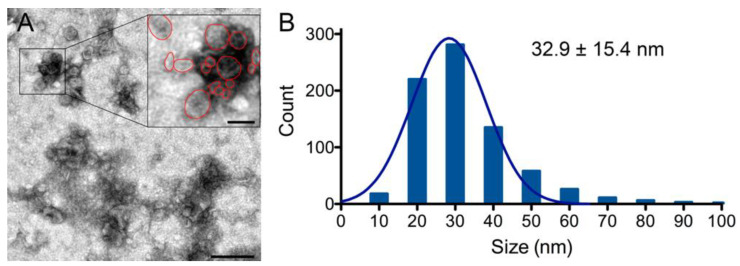
(**A**) Transmission electron microscopy depicts a representative transmission electron microscopy (TEM) image of PLT-Exos-Ca^2+^. Scale bar, 200 nm; scale bar in inset, 50 nm. (**B**) The bar graph shows the distribution diagram of the exosomes and the fitted Gaussian fit of the diameter values of exosomes as measured in the TEM images. The value on the upper right side of the graph represents the mean diameter ± SD of a total of 775 exosomes.

Biochemical characterization of platelet-derived exosomes by immunoblot using specific markers and antibody array analysis of more than 100 proteins showing high purity of exosomes isolated from calcium-stimulated platelets prompted us to analyze PLT-Exos-Ca^2+^ by transmission electron microscopy (TEM). As shown in Figure 5A, the vast majority of the negatively stained particles on TEM images exhibited the typical cup-shaped morphology described for exosomes. Assessment of particle size on TEM images revealed that more than 65% of particles had a diameter ranging from 20 to 40 nm. Particle distribution showed a single Gaussian population with a peak at 28.4 ± 9.8 nm and a mean diameter of 32.9 ± 15.4 nm (Figure 5B).

## 3. Discussion

Multiple studies support the role of PRP in regenerative medicine through its ability to modulate several processes, such as inflammation or cell activation, that influence tissue repair [28]. This is mainly due to the growth factors and cytokines released from platelets after their activation [29]. However, in addition to releasing free proteins into the affected environment, it is also known that platelets release proteins encapsulated in extracellular vesicles (EVs) known as exosomes. Similar to PRP, exosomes exert restorative effects [12,13], although their contribution to the healing and regenerative capacity of PRP is still unknown. Molecular characterization of these components of the platelet secretome may provide relevant information in this regard and new insights into the molecular substrate underlying their biological effects.

An important aspect when analyzing exosomes is to isolate them from a single origin, without contamination with those from other sources. With that purpose, we isolated exosomes exclusively released by PRP platelets, which were free of contaminants from other components present in PRP, allowing us to analyze pure PLT-Exos and to evaluate the influence of platelet activation by CaCl_2_ on their molecular composition. It must be taken into consideration that the activation method chosen may modify the molecular release [30]. The activation method employed here based on CaCl_2_ addition, is one of the most commonly used procedures to induce the release of molecular mediators from PRP platelets and avoids the use of exogenous biomolecules such as thrombin. At the same time, CaCl_2_-activation prevents local hypocalcemia caused by calcium chelating anticoagulants required for blood collection in the PRP preparation. A recent study showed that calcium restitution is necessary to prevent cellular and tissue alterations due to the anticoagulants used in some PRP preparations [30].

Our results showed that calcium activation caused protein levels to increase in both platelets and PLT-Exos. Although these results could be expected, because calcium triggers the coagulation cascade and platelet content is released into the medium [31], it is important to note that protein levels were approximately 20 times higher in the platelet fraction than in the PLT-Exos fraction. Therefore, it is unlikely that the increase in the protein content of PLT-Exos after stimulation with CaCl_2_ contributes significantly to the total effect of PRP. Despite this apparent low contribution of PLT-Exos to the total impact of platelets in CaCl_2_-activated PRP, an exhaustive characterization is important to shed light on the potential therapeutic use of these platelet-derived exosomes.

Immunoblotting for β-actin showed no differences between PLT versus PLT-Ca^2+^ or between PLT-Exos versus PLT-Exos-Ca^2+^ samples run side by side, whereas the intensity of Coomassie blue staining was similar in all the four samples. However, Coomassie-staining revealed clear differences in the band profiles between the calcium-activation versus baseline conditions, confirming that calcium impacts on the composition of the cargo release profile of PLT-Exos. To compare the presence of non-exosome and exosome-specific proteins in the different fractions, we chose a set of markers according to widely accepted criteria [11,12,13,14,25,32,33]. Interestingly, vesicles isolated from calcium-activated platelets were virtually devoid of plasma membrane (caveolin-1), endoplasmic reticulum (calreticulin), mitochondria (Human Porin proteins VDAC1 and 3, cyclophilin F, cytochrome C), lysosome (lamp-1), and early endosome (Rab5) markers, whereas those isolated from unstimulated platelets exhibited clear immunoreactivity for all of these proteins. These results indicate that PLT-Exos-Ca^2+^ fraction had higher purity in exosome vesicles than PLT-Exos, probably due to the fact that the number of exosomes released from platelets in the presence of calcium is greater than in its absence in basal conditions. In this sense, in a previous study, via Nanoflow analysis, it was found that the activation of human PRP by calcium gluconate yielded a higher concentration of exosomes particles in the exosomes-enriched fraction compared to the saline-activated group [14]. Analysis of exosome-specific markers [32,33] revealed that both type of exosomes exhibited immunoreactivity for the tetraspanin family of transmembrane proteins CD63, CD81, and CD9, along with the chaperone Hsp70 and the lipid raft marker flotillin-1. However, the relative intensity of the signal for these markers differed considerably, with PLT-Exos-Ca^2+^ exhibiting higher immunoreactivity for CD63 and CD81 antigens compared to PLT-Exos and weaker immunoreactivity for CD9, Hsp70, and flotillin-1. Of note, CD9-, hsp70- and flotillin-1 signals were even much weaker in PLT-Exos-Ca^2+^ than in activated and non-activated platelets. Strikingly, CD9-immunoreactivity was particularly strong in PLT-Exos but very weak in PLT-Exos-Ca^2+^, which may seem contradictory to the broad consensus that CD9, along with CD63 and CD81, is a reliable marker of exosomes in general [32,33] and platelet-derived exosomes in particular [11,12,13,14,25,34]. However, recent studies have provided strong evidence that only CD63 and CD81, but not CD9, are exosome-specific. Thus, in an untargeted proteomic analysis by liquid chromatography with tandem mass spectrometry (LC-MS/MS), Smolarz et al. [35] drew attention to the fact that EVs isolated from human serum and plasma often contain contaminants such as lipoproteins and a variety of serum components, and demonstrated that exosomes isolated by size exclusion chromatography from human serum are enriched for the exosome markers CD63 and CD81, but not for CD9. In the same line of evidence, a recent analysis of the origin of CD9- and CD63-bearing extracellular vesicles released from HeLa cells [36] showed that EVs enriched for CD9 and CD81 with little CD63 bud mainly from the plasma membrane as ectosomes, whereas those bearing CD63 and little CD9 form in internal compartments and qualify as exosomes. Finally, in an proteomic characterization of EVs purified from dendritic cells by a refined method that includes a density gradient ultracentrifugation step, it was concluded that Hsp70 and flotillin-1 are not exosome-specific markers [37].

Array analysis of cytokines, chemokines, and growth factors revealed higher levels in PRP relative to PPP for 12 of the 20 analytes detected. Noteworthily, in line with the proposed role of growth factors as major contributors of the positive effects of PRP in a variety of clinical conditions [3,38,39], EGF, PDGF-AA, PDGF-AB/BB, and BDNF were among the five analytes showing the highest fold-increase in PRP over PPP. The CXCL5 chemokine, whose levels were three times higher in PRP than in PPP was also found within this group of analytes. In the context of wound healing, CXCL5 is involved in the differentiation and recruitment/migration of bone marrow-derived MSCs [40], in late corneal healing phases [41], in the prevention of retinal ganglion cell death and promotion axonal regeneration [42], and in the enhancement of neurite outgrowth [43]. Followed by these six molecules, leptin levels increased almost by two-fold in PRP. Although little or no attention has been paid to this molecule in relation to the effects of PRP, this hormone promotes osteoblast proliferation while inhibiting osteoclast formation and activity at picomolar concentrations [44]. Furthermore, leptin stimulates chondrogenesis in damaged femoral cartilage [45], possibly being a relevant mediator of the effects of PRP in bone and cartilage. In addition to the aforementioned increase in analyte levels, significant lower levels were found in PRP than in PPP for a subset of only six analytes. Although we do not know the specific mechanisms by which the activation of PRP can result in a decrease in these levels, it is tempting to speculate that platelet proteases could account for this observation. In accordance with this interpretation, IGFBP-3, whose levels showed the greatest decrease in PRP compared to PPP, has been experimentally identified as a substrate for calpain-2 [46], which together with calpain-1 is a protease expressed by platelets that can be effectively activated by the 18 mM CaCl_2_ concentration [47] used for PRP activation. Regardless of the involvement of calpains, other proteases known to be released by platelets (e.g., MMP-9 [48] and ADAM DEC1 [49] metalloproteinases) or unknown mechanisms, upregulation or elevated serum levels of the four mediators that decreased most strongly in PRP relative to PPP have been associated with a variety of pathological effects and conditions such as TNF-α-induced proinflammatory responses in Alzheimer’s disease (lipocalin-2 [50]), diabetic peripheral neuropathy and cardiovascular risk (cystatin-C [51,52]), suppression of bone formation by inducing fibrosis (chitinase 3-like 1 [53]), or suppression of osteogenic differentiation and mineralization of adipose-derived stem cells (IGFBP-3 [54]). Altogether, these results further reinforce the already proven suitability of the preparation method used here to obtain a highly effective PRP for regenerative therapy [5,6,26].

Antibody array analysis revealed marked differences in the protein profile between plasma (PPP and PRP) versus exosome (PLT-Exos and PLT-Exos-Ca^2+^) samples, with several analytes showing strong signals in plasma while being undetectable or barely above the detection threshold in exosome samples. Indicative of the suitability of the procedure we used to isolate platelet-derived exosomes, of the 20 analytes detected here by antibody array analysis in exosome samples, six are among those previously found by untargeted LC-MS/MS (PF4, Apo A-I, VDB, Angiogenin, Complement C5/C5a, Adiponectin) in perhaps the most accurate proteomic characterization of human serum exosomes [35]. In the same sense, of the 15 molecules detected here in PPP or PRP, but not in exosomes, only one was found in the mentioned LC-MS/MS analysis. Further, of the six molecules concurring in both studies, four (PF4, Apo A-I, VDB, Angiogenin) were among the five exhibiting stronger intensities in our analysis of exosome samples and, interestingly, five (PF4, Apo A-1, VDB, Complement C5/C5a, Adiponectin) ranked consistently higher in PLT-Exos-Ca^2+^ than PLT-Exos, despite an overall trend for most signals toward a decrease in PLT-Exos-Ca^2+^ relative to PLT-Exos. It is also worth noting that the signal for PF4, which is the most abundant protein in human serum exosomes and a reliable exosome marker in matrix-assisted laser desorption/ionization mass spectrometry (MALDI-MS) [55], ranked first in PLT-Exos-Ca^2+^ but not PLT-Exos. Ultimately, the data obtained by antibody array, in combination with the results discussed above on the relative abundance of classical exosome-specific and non-exosome markers, further reveal that PLT-Exos-Ca^2+^ are highly enriched in exosome vesicles and definitely validate the isolation procedure for obtaining highly enriched preparations of platelet-derived human exosomes. In this regard, although other more elaborate methods for exosome isolation could produce purer samples than those obtained by the method used here, it is important to note that these procedures would be difficult to apply in clinical practice, not only for technical reasons but also because the additional intermediate steps and reagents would make it difficult to ensure sterile conditions with loss of yield and perhaps little gain in terms of purity.

Another major conclusion that can be drawn from our data showing that PLT-Exos-Ca^2+^ represent less than 0.3% of the total protein in activated PRP is that exosomes contribute negligibly to the clinical effects attributed to PRP. Nonetheless, purified platelet-derived exosomes can be potent promoters of different biological responses if administered in sufficient doses, and quantity is not a limiting factor in the case of human platelet exosomes. In fact, recent studies have provided evidence that PLT- or PRP-exosomes promote a variety of regeneration-related cellular and tissue responses such as re-epithelialization of chronic skin wounds [12], inhibition of apoptosis in osteonecrosis and osteoarthritis [11,13], fibrogenic activity in retinal Muller cells [56], or induction of dermal hair papilla cells [57], and to exert positive effects in the treatment of muscle injuries [58] and degeneration of the intervertebral disc [59]. Probably driven by the first work that described elevated levels of PDGF, bFGF, VEGF, and TGF-β in platelet-derived exosomes [10], most of these studies have focused on the high levels of these four growth factors as the main mediators of the efficacy of the exosomes in regeneration [11,12,13,56]. In apparent contradiction to that described by these authors, in our assay we only detected PDGF (PDGF-AA and PDGF-AB/BB) in exosome samples. Surely, the different sensitivity between the Western blot assays used by the authors and the antibody array used here could explain these discrepancies. Although weaker signals were observed for PDGF-AA, PDGF-AA/BB, and BDNF in either of the two exosome samples than in PRP, the signals are not comparable to determine the differences between both samples in the abundance of analytes due to the fact that 140 times more plasma protein was added to the assay than exosomes. However, with these data we can presume stronger signals from the mentioned growth factors in PLT-Exos and PLT-Exos-Ca^2+^ than in PRP if the total protein amounts were equivalent, which is not practicable in our approach. Indeed, previous studies reporting higher levels of growth factors in platelet- or PRP-derived exosomes than in PRP [11,12,14,56] or analyzing their efficacy against PRP [10,11,12,13] used equal amounts of total PRP and exosome protein. Strikingly, lower levels of PDGF-AA, PDGF-AA/BB, and BDNF were observed in PLT-Exos-Ca^2+^ compared to PLT-Exos. Based on the pieces of evidence discussed here that reveal a higher purity of PLT-Exos-Ca^2+^, a possible interpretation could be given by the presence of growth factors associated with non-exosome particles in PLT-Exos, which would not be present in PLT-Exos-Ca^2+^. Alternatively, it is possible that calcium affects the content of specific exosome cargo proteins.

From the data discussed so far, we conclude that the PLT-Exos isolated from CaCl_2_-activated platelets described here exhibit high purity and meet the most up-to-date biochemical criteria that characterize exosomes against other EVs and contaminants. Furthermore, morphological analysis by TEM analysis of the negatively stained particles revealed a typical cup-shaped exosome morphology and a diameter distribution showing a single Gaussian population. However, the mean diameter observed here for PLT-Exos isolated from activated platelets (32.9 ± 15.4) was considerably smaller in relation to the values reported in previous studies. For example, Rui et al. [14] described a mean diameter of 90.32 ± 54.65 nm for exosomes obtained by centrifugation methods from unstimulated PRP, and of 94.4 ± 46.1, 78.0 ± 35.9, and 84.8 ± 34.1 nm for exosomes obtained from PRP stimulated by calcium-gluconate, thrombin, and a combined calcium-gluconate/thrombin, respectively. For their part, Liu et al. [13] described a mean diameter of 145.6 ± 50.4 nm for exosomes extracted from un-stimulated PRP with a commercial kit. However, the high-quality images shown by Heijenen et al. in a seminal report on platelet-derived exosomes [25] show a size for PLT-Exos isolated from platelet pellets similar to that described here. In fact, although the authors of this report did not provide mean size data, we analyzed the size of particles shown in their Figure 2B, obtaining a mean size value of 34.9 ± 8.6 nm, which is virtually identical to the here reported size for PLT-Exos. The discrepancies in the calculated size in different studies could be due, at least in part, to methodological differences in the exosome isolation procedure, including the PRP used and, more importantly, the source from which exosomes were purified. It should be noted that Rui et al. [14] and Liu et al. [13] used PRP as a source for the isolation of exosomes and, therefore, it is conceivable that their preparations contained a heterogeneous mixture of exosomes of various cellular origins. Rather, in the same way that we did here, Heijenen et al. [25] used activated platelets washed from PRP as a source for the isolation of exosomes, which could explain the great agreement between our and their results.

The main limitations of the present study are the sample size and the fact that it was circumscribed to a single platelet activation method. Thus, it would be interesting to carry out further analyses that include different activation methods such as thrombin or collagen. In any case, our results show that platelets produce more purified exosomes and with higher yields under calcium activation than under basal conditions. Further studies will be necessary to determine whether these exosomes have an intrinsic therapeutic potential, as well as to evaluate their suitability as a nanosystem that could be adapted, according to the desired biological effects, by loading therapeutic compounds or biomolecules to achieve a certain cargo profile or by surface functionalization to specifically target the desired biological targets.

## 4. Materials and Methods

### 4.1. Platelet-Rich Plasma Preparation and Characterization

The institutional review board approved this study and informed consent was obtained from every patient from whom biological samples were extracted (Code UCA-O8/EE/19/EXO, 3 March 2018).

**Figure 6:**
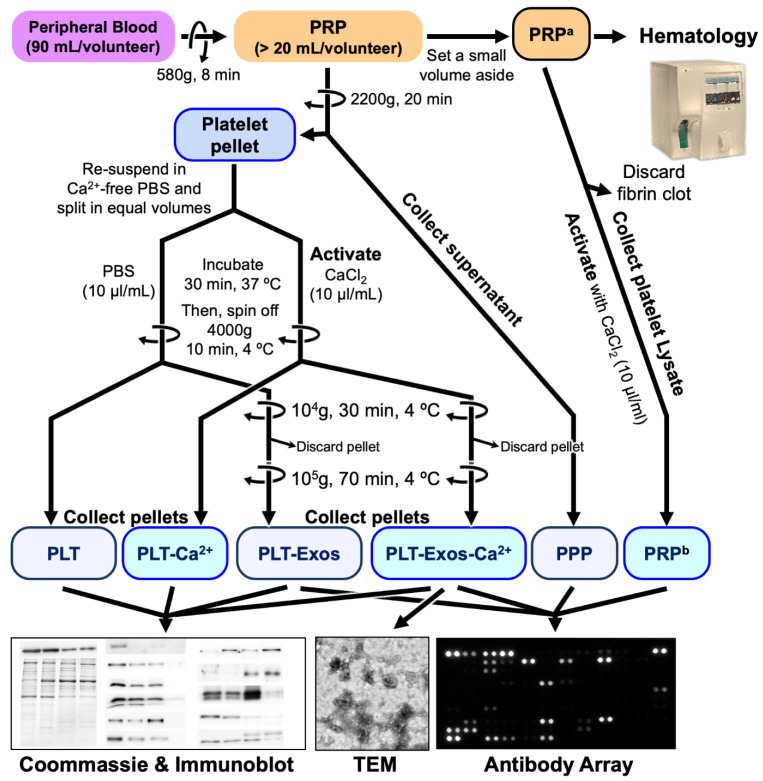
Flow diagram of the isolation of the different samples used in the present study from whole human blood of young adults and the techniques used for their analysis. ^a^ It consists of the plasma fraction above the sedimented red blood cells, but not including the buffy coat. ^b^ This fraction, referred to as PRP throughout the text, corresponds to the platelet lysate collected from the activated PRP.

PRP was prepared from the peripheral blood of three healthy donors. First, 90 mL of venous blood was extracted from each donor in order to prepare the PRP and stored in 8-mL tubes containing 3.8% (*w*/*v*) sodium citrate anticoagulant solutions. Blood was centrifuged at 580× *g* for 8 min at room temperature. After centrifugation, the plasma fraction located above the sedimented red blood cells was collected in a tube without including the buffy coat, according to the protocol [5]. This process avoids the inclusion of white blood cells and reaches a moderate concentration of platelets (1 to 2 times the concentration of platelets compared with peripheral blood, depending on the platelet count and size as well as the hematocrit) and an absence of erythrocytes and leukocytes [26]. Collected PRP were assayed using a hematology analyzer to evaluate the erythrocyte, leukocyte, and platelet content (Figure 6).

### 4.2. Isolation of Human Platelet-Derived Exosomes Obtained under Basal Conditions and CaCl_2_ Stimulation

Platelet-derived exosomes were purified by differential ultracentrifugation based on the method described by Théry et al. [32], with minor modifications. A volume of 20 mL of human PRP collected from each volunteer was centrifuged at 2200× *g* for 20 min at room temperature (20 °C). The supernatant (Platelet-Poor Plasma, PPP) was aliquoted and stored at −80 °C, and the platelet-enriched pellet was resuspended in 20 mL phosphate buffered saline, pH 7.4 (PBS) and distributed in 1 mL aliquots. Half the aliquots were used to activate platelets by adding 20 µL of CaCl_2_ (10% *w*/*v*) per ml platelet-suspension as the source for the isolation of exosomes from calcium activated platelets (PLT-Exos-Ca^2+^). An amount of 20 µL PBS was added to the remaining aliquots as the source for the isolation of exosomes from non-activated platelets (PLT-Exos). Platelet suspensions were incubated for 30 min at 37 °C and centrifuged at 4000× *g* for 10 min at 4 °C. The calcium-activated and non-activated platelet pellets were stored at −80 °C, and the supernatant was centrifuged at 10,000× *g* for 30 min at 4 °C. The pellet containing cell debris was discarded, and the exosomes-containing supernatant was subjected to an ultracentrifugation step at 100,000× *g* for 70 min at 4 °C (SW 40 Ti Swinging-Bucket Rotor, Beckman coulter) to obtain the exosome pellet, which was re-suspended in PBS, aliquoted, and stored frozen at −80 °C. Protein content was determined by the Bradford method using Bio-Rad Protein Assay (Bio-Rad Laboratories, Madrid, Spain), according to the manufacturer’s protocol, with bovine γ-globulin as standard. The exosome purification procedure and the purpose of each of the samples is shown in Figure 6.

### 4.3. Western Blot Analysis

Western blot studies were performed as previously reported, with minor modifications [60,61,62]. Briefly, samples were heated at 95 °C in urea-denaturing buffer (20 mM Tris-HCl, pH 8.0, 12% glycerol, 12% urea, 5% dithiothreitol, 2% sodium dodecyl sulfate, 0.01% bromophenol blue) over a period of 10 min, and 5 µg (β-actin) or 20 μg (the rest of antigens) protein were resolved by electrophoresis in 10% SDS-polyacrylamide gels (SDS-PAGE) using the Mini Protean II gel apparatus (Bio-Rad; Hercules, CA, USA). Then, gels were either stained with Coomassie blue or used to transfer resolved proteins to polyvinylidene fluoride (PVDF) membranes (Amersham Biosciences, Piscataway, NJ, USA) using the Mini TransBlot transfer unit (Bio-Rad; Hercules, CA, USA) at 30 V overnight at 4 °C. For immunoblotting, PVDF membranes were blocked in PBS containing 5% non-fat dry milk, 0.5% BSA, and 0.2% Tween-20 for 1 h (blocking buffer), and incubated overnight at 4 °C (in blocking buffer without milk) with primary antibodies (Table 3). Blots were then washed and incubated for 2 h at room temperature with horseradish peroxidase (HRP) conjugated donkey anti-Rabbit IgG (NA934; Amersham Biosciences) or HRP-conjugated Sheep anti-Mouse IgG (NXA931; Amersham Biosciences) secondary antibodies, all diluted to 1:10,000 in blocking buffer. Immunoreactive bands were visualized with Clarity Western ECL Substrate (#1705061; Bio-Rad Laboratories) according to the manufacturer instructions. A broad-range pre-stained protein ladder (1610373EDU, Bio-Rad) was used to estimate the molecular mass of individual bands.

### 4.4. Transmission Electron Microscopy and Analysis of Exosome Size

Samples of PLT-Exos-Ca^2+^ from volunteer 3 were used for this analysis. For each preparation to be stained, two microliters sample were adhered for 1 min onto glow-discharged Carbon coated grids (Agar S160-3 grids and Leica ACE200-glow discharge for 30 s at 10 mA). After removing excess liquid by blotting with Whatman filter paper, grids were negatively stained using 1% uranyl acetate for 1 min. Preparations were visualized in a JEOL JEM 1400 Plus transmission electron microscope operated at 100 kV, and images were acquired with an integrated Hamamatsu Flash sCMOS digital camera.

Measurements of the mean diameter of exosomes was performed on two TEM images (3.1 μm^2^ each) from two different samples. First, using the vector drawing tool of Adobe Photoshop Adobe 22.5.0 (San Jose, CA, USA), the perimeters of all the exosomes identified in the micrographs were manually outlined. For very close or partially overlapping particles, perimeters were outlined in separate layers to avoid contact between contours. Subsequently, the outlines were filled in with the Adobe Photoshop paint bucket tool, and separate layers were saved individually and imported as a stack into Fiji-ImageJ 1.53f51 (NIH, Bethesda, MA, USA). The images were then converted to binary in Fiji-ImageJ, and the Feret’s diameters were measured using the Analyze Particles macro (NIH, Bethesda, MA, USA).

### 4.5. Analysis of Cytokines, Chemokines and Growth Factors

Molecules and cytokines were analyzed using the Proteome profiler human XL Cytokine array kit (ARY022B, R&D Systems, Minneapolis, MN, USA). Each of the types of samples to be analyzed was prepared by mixing equal amounts of protein from volunteers 1 and 2. PPP and PRP (both at 117 µg/µL) were prepared for the assay by diluting 10.5 mg (90 µL) of either sample in a final 1.5 mL volume of the resuspension buffer (buffer 6), as recommended by the supplier for serum samples. To ensure the release of protein cargo from exosomes, an amount of PLT-Exos and PLT-Exo-Ca^2+^ samples containing 75 µg total protein was first lysed using a lysis buffer recommended by the supplier for tissue lysates (0.5% Igepal, 0.5% sodium deoxycholate, 0.1% sodium dodecyl sulfate, and 150 mM NaCl in 50 mM Tris-HCl, pH 7.5) and then diluted to a final 1.5 mL volume of resuspension buffer 6. Thereafter, PPP, PRP, PLT-Exos, and PLT-Exo-Ca^2+^ samples were assayed side by side in strict accordance with the manufacturer’s instructions. After the last incubation step in streptavidin-HRP reagent, membrane antibody arrays were processed for chemiluminescence with the reagents provided in the kit. Signals were acquired using an ImageQuant 350 imager (GE Healthcare, Madrid, Spain) and quantified by densitometry using ImageJ image analysis software (NIH, Bethesda, MA, USA).

### 4.6. Data Processing, Statistical Analysis, and Figure Construction

Considering the volume of PRP from which the platelets of each volunteer were washed to obtain exosomes under basal conditions and after activation by CaCl_2_, the protein yield in non-activated and activated platelets and in PLT-Exos and PLT-Exos-Ca^2+^ was normalized to the initial platelet count in PRP and expressed as ng protein/10^6^ platelets. Data were entered into GraphPad Prism 5.0 (San Diego, CA, USA) for statistical analyses using one-tailed Student’s *t*-test. Effect size of Ca^2+^-stimulation on protein amount in platelets and PLT-Exos was estimated by Hedges’ *g* using the equation $Hedges^{\prime }g=\left(M1-M2\right)/\left(Weighted\;Sp\right)$ [63], where *M1 − M2* is the difference in mean and *Weighted Sp* is the pooled and weighted standard deviation. The mean diameter of PLT-Exos-Ca^2+^ was calculated from a total of 775 values. GraphPad Prism 5.0 (San Diego, CA, USA) was used to generate the frequency distribution histogram of vesicle diameter and the corresponding fitted Gaussian curve. Integrated optical density (OD) values obtained in duplicate for each of the analytes included in the Proteome profiler human XL Cytokine array kit (R&D Systems) were graphically represented through Microsoft Excel (Microsoft Corporation, Redmond, Washington, USA). All statistical analyses were performed with GraphPad Prism 5.0 and are detailed in the figure legends corresponding to the different assays. Statistical significance was set at 95% confidence level (*p* < 0.05). Graphs were generated with GraphPad Prism and Microsoft Excel and were saved as tiff files, and figures were assembled with Adobe Photoshop 22.5.0.

## 5. Conclusions

The present work reveals that the calcium activation of PRP promotes the release of highly purified platelet-derived exosomes, showing a concordant size and morphology and absence of contaminants from other cellular compartments. Furthermore, calcium was proved to alter the cytokine cargo expression profile of PLT-Exos-Ca^2+^ that differs markedly in relation to exosomes isolated from non-activated platelets. Importantly, although PRP calcium activation promotes exosome release, its net contribution to the total PRP effect was found to be minimal in view of the low yield of exosome protein relative to total PRP protein.

In brief, although further studies with more donors are needed to confirm these initial findings, the results provide new insights into the biochemical nature of platelet-derived exosomes, unveil the contribution of the exosome component to the clinical efficacy of PRP, and provide a basis for future trials to assess the potential of these nanovesicles in regenerative therapy and drug delivery.

## Figures and Tables

**Table 1:** Summary of Platelet-Rich Plasma characteristics.

1. PRP Preparation	
Initial blood volume	90 mL per subject (8 mL per tube)
Anticoagulant	Sodium citrate 3.8% (*w*/*v*)
System	Close
Centrifugation	Yes
Number	1
Seed	580 g–8 min
Final PRP volume	>20 mL per subject
**2. PRP Characteristics**	
PRP Type	13-00-11
MPV	8.8 ± 0.6 fL
Red Blood Cells	<0.01 × 10^6^/µL
White Blood Cells	<0.05 × 10^6^/µL
*Neutrophils*	-
*Lymphocytes*	-
*Monocytes*	-
*Eosinophils*	-
*Basophils*	-
Activation	10 µL CaCl_2_ (10% *w*/*v*) per mL PRP
**3. Other Remarkable PRP and Study features**	
The product used for the analysis of cytokine and growth factors was the platelet lysate obtained after activation of PRP with CaCl_2_.	

**Table 2:** Analyte values.

N.	Coordinates	Analyte	Integrated OD ± SD				Integrated OD ± SD			
			PPP	PRP	*p* Value	PRP/PPP Ratio	Exo	Exo-Ca^2+^	*p* Value	Exo-Ca^2+^/Exo Ratio
1	A3, A4	Adiponectin	1114 ± 5	1017 ± 45	0.094	0.91	289 ± 41	227 ± 22	0.200	0.79
2	A5, A6	Apo A-I	**1817 ± 88**	**2280 ± 53**	0.024 *	**1.26**	**1892 ± 38**	**1554 ± 35**	0.011 *	**0.82**
3	A7, A8	Angiogenin	2840 ± 12	2720 ± 102	0.242	0.96	898 ± 250	571 ± 5	0.206	0.66
4	A15, A16	BDNF	**256 ± 1**	**597 ± 45**	0.009 **	**2.33**	**350 ± 22**	**193 ± 10**	0.012 *	**0.55**
5	A17, A18	Complement C5/C5a	**474 ± 35**	**695 ± 16**	0.015 *	**1.47**	**143 ± 17**	**359 ± 9**	0.004 **	**2.52**
6	B5, B6	Chitinase 3-like 1	**981 ± 71**	**567 ± 18**	0.015 *	**0.58**				
7	B7, B8	Complement Factor D	444 ± 26	452 ± 4	0.720	1.02				
8	B9, B10	C-Reactive Protein	2287 ± 80	2059 ± 48	0.074	0.90				
9	B13, B14	Cystatin C	**1388 ± 134**	**876 ± 11**	0.033 *	**0.63**				
10	B17, B18	Dipeptidyl peptidase-4 (DPP4)	2632 ± 216	2486 ± 45	0.449	0.95				
11	B19, B20	EGF	**108 ± 16**	**395 ± 11**	0.002 **	**3.70**				
12	C3, C4	Emmprin	180 ± 23	131 ± 36	0.246	0.74	**328 ± 19**	**42 ± 7**	0.003 **	**0.13**
13	C3, C4	CXCL5 (ENA-78)	**183 ± 4**	**613 ± 91**	0.022 *	**3.35**	**116 ± 7**	**39 ± 13**	0.018 *	**0.34**
14	C5, C6	Endoglin	895 ± 46	851 ± 35	0.394	0.95	**70 ± 5**	**9 ± 7**	0.010 *	**0.13**
15	D7, D8	ICAM-1	**314 ± 23**	**457 ± 38**	0.044 *	**1.46**				
16	D11, D12	IGFBP-2	1470 ± 80	1434 ± 104	0.735	0.98				
17	D13, D14	IGFBP-3	485 ± 48	250 ± 22	0.025 *	**0.52**				
18	E23, E24	IL18-Binding Protein	839 ± 155	1034 ± 157	0.338	1.25				
19	G1, G2	Leptin	**234 ± 40**	**415 ± 5**	0.024 *	**1.80**				
20	G5, G6	Lipocalin-2	**601 ± 30**	**403 ± 3**	0.011 *	**0.67**	96 ± 4	70 ± 4	0.193	0.73
21	G23, G24	MMP-9	209 ± 32	369 ± 47	0.057	1.78				
22	H3, H4	Osteopontin	670 ± 40	573 ± 39	0.135	0.86				
23	H5, H6	PDGF-AA	**247 ± 27**	**544 ± 11**	0.005 **	**2.22**	**220 ± 28**	**73 ± 7**	0.019 *	**0.34**
24	H7, H8	PDGF-AB/BB	**156 ± 32**	**404 ± 12**	0.010 **	**2.65**	**194 ± 14**	**47 ± 7**	0.006 **	**0.24**
25	H11, H12	Platelet factor 4 (PF4)	1889 ± 162	2367 ± 172	0.103	1.26	2004 ± 84	2135 ± 272	0.642	1.07
26	H15, H16	CCL5	427 ± 78	500 ± 81	0.455	1.19	731 ± 57	531 ± 64	0.081	0.73
27	H17, H18	RBP-4	**2000 ± 7**	**1861 ± 32**	0.028 *	**0.93**	**799 ± 34**	**544 ± 68**	0.042 *	**0.68**
28	I1, I2	PAI-1	1548 ± 275	1860 ± 84	0.265	1.22	411 ± 9	564 ± 50	0.051	1.37
29	I3, I4	SHBG	**1560 ± 15**	**1949 ± 35**	0.005 **	**1.25**	**279 ± 32**	**23 ± 1**	0.008 **	**0.08**
30	I9, I10	Trefoil Factor 3 (TFF3)	146 ± 39	156 ± 17	0.780	1.11	667 ± 48	513 ± 37	0.069	0.77
31	I15, I16	Thrombospondin-1	703 ± 39	957 ± 77	0.053	1.36				
32	J5, J6	Vitamin D BP (VDB)	**1740 ± 47**	**2126 ± 55**	0.017 *	**1.22**	1809 ± 115	1520 ± 2	0.070	0.84
33	J7, J8	PECAM-1 (CD31)	**977 ± 1**	**828 ± 20**	0.009 **	**0.85**	**2070 ± 2**	**784 ± 26**	0.0002 ***	**0.38**
34	J9, J10	TIM-3	345 ± 75	304 ± 18	0.529	0.90	109 ± 40	40 ± 13	0.124	0.38
35	J11, J12	VCAM-1	**1662 ± 38**	**2035 ± 59**	0.017 *	**1.22**				

Exo, exosomes isolated from non-activated platelets, Exo-Ca^2+^, exosomes isolated from CaCl_2_-activated platelets. Values corresponding to analytes with significant differences between PRP versus PPP and Exo versus Exo-Ca^2+^ conditions are in bold. * *p* < 0.05, ** *p* < 0.01, *** *p* < 0.005. *p*-values were calculated by Student’s unpaired (two-tail) *t*-test. For comparative purposes between plasma (PPP and PRP) and exosome samples (PLT-Exos and PLT-Exos-Ca^2+^), note that 140 times more protein from PPP or PRP (10.5 mg) than from PLT-Exos or PLT-Exos-Ca^2+^ (75 μg) was added to the assay.

**Table 3:** Primary antibodies used.

Target	Dilution	Clonality (Clone)	Species (Isotype)	Immunizing Antigen	Use	Source, Catalog N.
β-actin	1:5000	Monoclonal (AC-15)	Mouse (IgG1)	Epitope mapping at the N-terminal end of β-actin	Loading control	Sigma, A5441,
Caveolin-1	1:750	Polyclonal	Rabbit (IgG)	Synthetic peptide mapping at the N-terminus of caveolin-1	Components of cell surface machinery for endosome formation	Santa Cruz Biotech., sc-894
Human Porin proteins VDAC1,3	1:1000	Monoclonal (20B12AF2)	Mouse (IgG2b)	Full length protein	Mitochondrial marker	Abcam, ab14734
Cyclophilin F	1:1000	Monoclonal (E11AE12BD4)	Mouse (IgG1)	Full length Rat Cyclophilin F	Mitochondrial marker	Abcam, ab110324
Cytochrome C	1:1000	Monoclonal (37BA11)	Mouse (IgG2a)	Bovine heart Cytochrome C	Mitochondrial marker	Abcam, ab110325
Calreticulin	1:1000	Monoclonal (FMC 75)	Mouse (IgG)	Fusion protein comprising calreticulin	Endoplasmic reticulum marker	Abcam, ab22683
Lamp1	1:500	Monoclonal (H4A3)	Mouse (IgG1)	Adherent spleen cells	Lysosome marker	Santa Cruz Biotech., sc-20011
Rab5	1:1000	Monoclonal (D-11)	Mouse (IgG2b)	Full-length Rab5A	Early endosome marker	Santa Cruz Biotech., sc-46692
Flotillin-1	1:1000	Polyclonal	Rabbit (IgG)	Residues 1-100 of human flotillin-1 conjugated to KLH	Lipid raft marker, often present in exosome membranes	Abcam, ab41927
Hsp70	1:1000	Polyclonal	Rabbit (ns)	License protected	Platelet cytosolic protein identified as a cargo protein in some exosomes	System Biosciences, EXOAB-Hsp70A-1
CD63	1:3000	Polyclonal	Rabbit (ns)	License protected	Widely used as exosome surface marker	System Biosciences, EXOAB-CD63A-1
CD81	1:1000	Polyclonal	Rabbit (ns)	License protected	Widely used as exosome surface marker	System Biosciences, EXOAB-CD81A-1
CD9	1:1000	Polyclonal	Rabbit (ns)	License protected	Widely used as exosome surface marker	System Biosciences, EXOAB-CD9A-1

ns, not specified by the vendor.

## Data Availability

The data presented in this study are available within the article. Additional inquiries may be directed to the corresponding authors.
